# FVC/D_LCO_ identifies pulmonary hypertension and predicts 5-year all-cause mortality in patients with COPD

**DOI:** 10.1186/s40001-023-01130-6

**Published:** 2023-05-15

**Authors:** Yuer Li, Rui Zhang, Hu Shan, Wenhua Shi, Xiaoli Feng, Haijuan Chen, Xia Yang, Yali Li, Jie Zhang, Ming Zhang

**Affiliations:** Department of Respiratory and Critical Care Medicine, The Second Affiliated Hospital of Xi’an Jiaotong University, No.157 West Fifth Road, Xi’an, 710004 Shaanxi China

**Keywords:** Chronic obstructive pulmonary disease, Pulmonary hypertension, Mortality, Forced vital capacity, Diffusing capacity of carbon monoxide

## Abstract

**Background:**

Pulmonary hypertension (PH) is a common complication of chronic obstructive pulmonary disease (COPD). However, it is unknown whether the ratio of forced vital capacity (FVC) to diffusing lung capacity for carbon monoxide (D_LCO_) can identify PH in the patients with COPD and predict its prognosis.

**Methods:**

The study population I included 937 COPD patients who were admitted to inpatient treatments from 2010 to 2017, and finally 750 patients were available to follow-up the 5-year all-cause mortality (study population II). Clinical characteristics of the study population were recorded.

**Results:**

COPD patients with PH had a higher FVC/D_LCO_ value compared with the patients without PH. The threshold for FVC/D_LCO_ to identify PH in COPD patients was 0.44 l/mmol/min/kPa. Multivariate logistic regression analysis showed that FVC/D_LCO_ was a significant predictor for PH in the patients with COPD. The study population II showed that the 5-year all-cause mortality of COPD patients was significantly higher in combined with PH group than without PH group. Compared with the survivor group, FVC/D_LCO_ value was significantly increased in non-survivor group. The threshold for FVC/D_LCO_ to predict 5-year all-cause mortality was 0.41 l/mmol/min/kPa. Kaplan–Meier survival curves showed that 5-year cumulative survival rate for COPD patients were significantly decreased when the value of FVC/D_LCO_ was ≥ 0.41 l/mmol/min/kPa. Multivariate cox regression analysis showed that FVC/D_LCO_ was an independent prognostic factor for 5-year all-cause mortality in COPD patients.

**Conclusion:**

FVC/D_LCO_ could identify PH in the patients with COPD and was an independent predictor for 5-year all-cause mortality of COPD.

**Supplementary Information:**

The online version contains supplementary material available at 10.1186/s40001-023-01130-6.

## Introduction

Chronic obstructive pulmonary disease (COPD) is a preventable disease in which persistent respiratory symptoms and airflow limitations worsen over the time. It is the third leading cause of death in the world, and causes more than 3 million people deaths worldwide each year [1]. COPD is associated with several complications, including respiratory failure, pulmonary encephalopathy, cor pulmonary, lung cancer, weight loss and skeletal muscle dysfunction [2, 3]. It has been proved that these complications have significant impacts on COPD prognosis with increased death risk [4].

Pulmonary hypertension (PH), a common complication of COPD, is a pathophysiological disorder characterized by abnormally elevated pulmonary artery pressure which is defined by mean pulmonary artery pressure (mPAP) > 20 mmHg at rest [5]. It has been reported that mPAP in the patients with stable COPD developed slowly over the time [6]. Although only an average change of ± 0.4 mmHg/year [6], the prevalence of PH was significantly high in the end-stage of COPD patients, even up to 90% [7, 8]. Furthermore, PH is an essential factor to assess the prognosis of COPD patients [9]. Several studies have found that the presence of PH has adverse impacts on COPD patients, such as decreased exercise tolerance [9], increased hospitalization rates due to acute exacerbation of COPD [10], and reduced survival rate [11, 12]. It has also been well known that the decisive factor for the prognosis of COPD patients was pulmonary artery pressure, even received long-term oxygen therapy [13]. And Vizza et al. found that PH secondary to COPD patients had an even worse prognosis than idiopathic pulmonary hypertension [14]. With the increase of pulmonary pressure in COPD patients, they are more prone to develop right ventricular enlargement (hypertrophy and/or dilation), right heart failure and increased mortality [15]. Previous study has shown that many COPD patients with severe PH have an additional cause of pulmonary pressure elevation, such as left ventricular disease [16], pulmonary embolism [17] or sleep apnea syndrome [18], and severe PH seems responsible for notable exertional dyspnea and reduced survival in the patients with COPD [19]. Therefore, early recognition of PH is very important for judging the prognosis of COPD.

Right heart catheterization (RHC) is the gold standard for the diagnosis of PH [20], but this technique is an invasive test with associated risk of complications. Transthoracic Doppler echocardiography is recommended as the main noninvasive modality in the screening and evaluation on PH [21]. Nevertheless, pulmonary artery systolic pressure obtained by echocardiography was frequently underestimated, particularly when quality of the Doppler envelope was fair or poor [22]. In order to obtain reliable results, we should choose a specialized imaging physician to operate the Doppler echocardiography to reduce the errors caused by technical factors. Electrocardiogram and chest radiography can provide useful information for PH diagnosis, but these two examinations have lower sensitivities, and a negative result cannot exclude PH [23]. In addition, the clinical symptoms and signs of PH are not specific, such as exertional dyspnea, fatigue, chest pain, augmented second heart sound in the pulmonary valve area, and right ventricular failure (edema, ascites and hepatojugular reflux). Moreover, these symptoms and signs usually occur in the severe stage rather than the early stage of PH. Therefore, it is very important to find a simple and noninvasive tool to identify PH in the patients with COPD.

It has been reported that the value of forced vital capacity (FVC)/diffusing lung capacity for carbon monoxide (D_LCO_) was a predictor of PH in patients with systemic sclerosis [24]. Another study has demonstrated that D_LCO_% predicted < 55% was strongly associated with PH in systemic sclerosis [25]. However, it is still unclear whether FVC/D_LCO_ can identify PH in COPD patients and indicate the prognosis of COPD. Therefore, this study aims to explore the role of FVC/D_LCO_ in identifying PH and predicting 5-year all-cause mortality of COPD patients.

## Methods

### Subjects

**Fig. 1:**
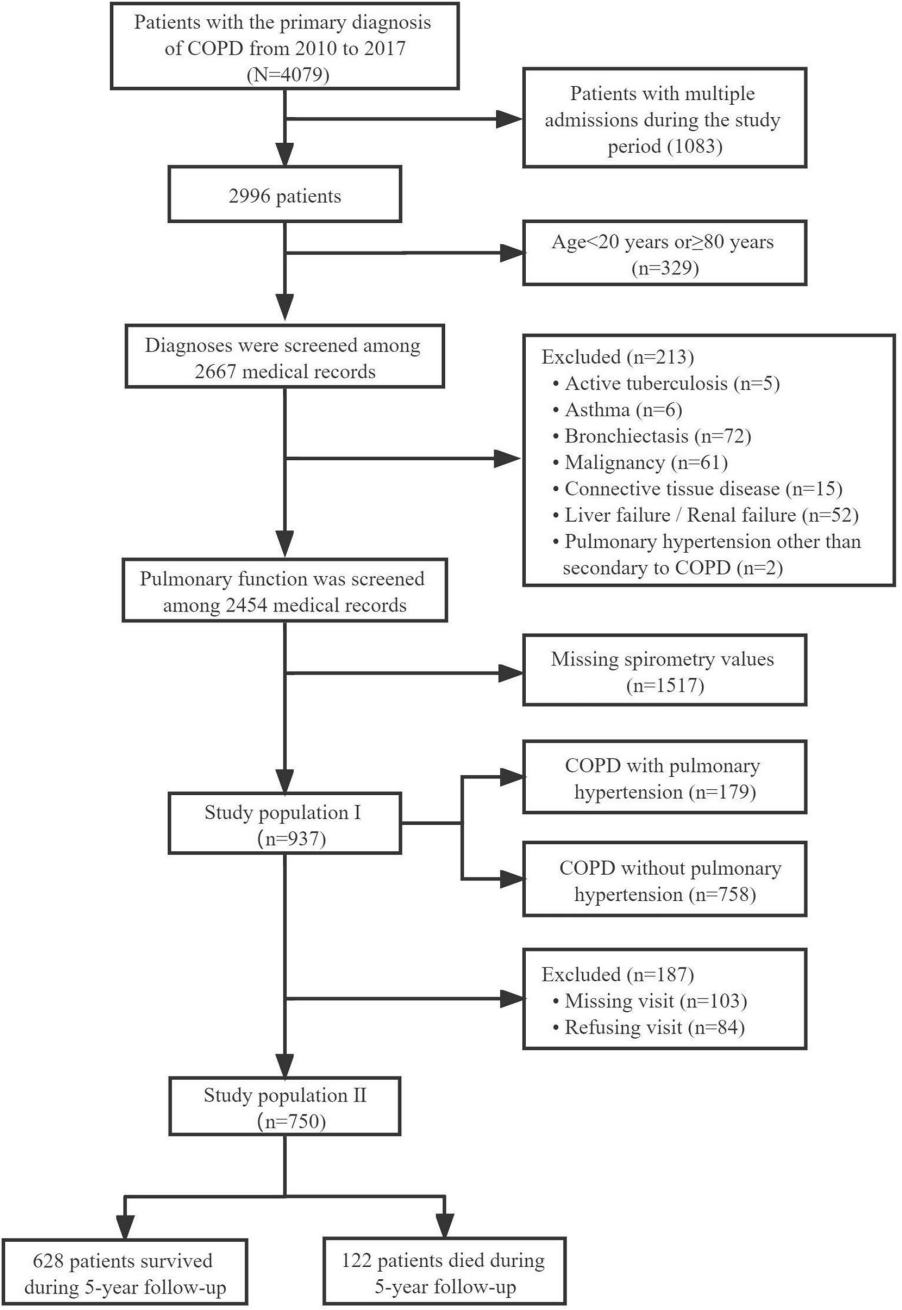
Flow chart of study patient

This is a single-center retrospective cohort study of COPD patients who received inpatient treatment due to the acute exacerbation at the Department of Respiratory and Critical Care Medicine, the Second Affiliated Hospital of Xi’an Jiaotong University, from 2010 to 2017. Only the first admission was recorded for patients with multiple admissions during the study period. This study excluded the patients aged < 20 years or ≥ 80 years. Patients with active tuberculosis, asthma, bronchiectasis, malignancy, connective tissue disease, liver failure, renal failure, or PH other than secondary to COPD were excluded from this study. After screening all medical records, 937 patients were included in the study population I in which 179 patients were diagnosed with PH secondary to COPD. The survival status of patients was retrospective follow-up for 5 years after leaving the hospital, and finally 750 patients were enrolled in the study population II, in which 122 patients died during the follow-up period (Fig. 1). COPD was defined as a post-bronchodilator forced expiratory volume in 1 s (FEV_1_)/FVC less than 0.70 [26]. PH was diagnosed by pulmonary artery systolic pressure > 35 mmHg determined by Doppler echocardiography using the modified Bernoulli equation [27]. All patients gave informed consent approved by the Research Committee of Human Investigation of the Second Affiliated Hospital of Xi’an Jiaotong University.

### Pulmonary function and blood gas analysis

Spirometry was performed for assessment of pulmonary function when the patients were stable enough to use the spirometer maneuver before leaving the hospital. Reversibility assessment was conducted in COPD patients with a short-acting beta-2 agonist (SABA). The arterial blood sample was immediately collected and analyzed when COPD patients were admitted to the hospital.

### Clinical and biochemical examinations

Demographic and clinical information of all participants were recorded in detail. Smoking history, history of disease and survival time were also collected. Routine blood test, D-dimer, liver function and renal function were usually determined at the beginning of hospitalization, and all these parameters were collected in this study.

### Statistical analysis

All data were examined with Kolmogorov–Smirnov test for normal distribution. Normally distributed data were presented as means ± standard deviation (SD). Non-normally distributed data were presented as median (interquartile range). Categorical variables were presented as percentages. The comparison between two groups were used the Student’s t test, Mann Whitney U test or Chi-square test according to the data type. The receiver operating characteristic (ROC) curve was used to determine the FVC/D_LCO_ threshold. Later the logistic regression model was used to explore the factors associated with PH in COPD patients. The factors of all-cause mortality were analyzed using the COX regression model. All variables detected in the univariate analyses (with a P-value less than 0.05) were included in the multivariate analysis. Survival curves were drawn by the Kaplan–Meier method, and 5-year all-cause mortality was compared between the elevated and non-elevated FVC/D_LCO_ groups. A value of P < 0.05 was considered significant. Statistical analyses were conducted with SPSS version 17.0 software (SPSS Inc., Chicago, IL, USA).

## Results

### Characteristics of the patients in the study population I

The clinical and physiological characteristics of study population I are presented in Table 1, and about 19.1% of COPD patients occurred PH. COPD patients with PH were older, and had lower BMI and higher smoking index compared with the patients without PH (all P < 0.05). When the COPD patients left the hospital, 83.6% of patients had received the inhalation therapy, including short-acting bronchodilator (SABD), inhaled corticosteroids (ICS)/long-acting beta-2 agonist (LABA), long-acting muscarinic antagonists (LAMA) and ICS/LABA + LAMA. However, there were no significant differences in these four inhalation therapies between the COPD patients with or without PH. The pulmonary function in COPD patients with PH was even worse than that in the COPD patients without PH (P < 0.05). Compared with COPD patients without PH, the value of FVC/D_LCO_ was significantly increased in the COPD patients with PH [0.51 (0.41–0.76) vs. 0.42 (0.34–0.53), P < 0.001]. And COPD patients with PH had a lower VA value (% predicted) compared with the patients without PH (P < 0.001). Moreover, partial pressure of oxygen in arterial blood (PaO_2_) significantly decreased and partial pressure of carbon dioxide in arterial blood (PaCO_2_) significantly increased in COPD patients with PH compared with COPD patients without PH (both P < 0.001). However, alveolar-arterial oxygen gradient [PO_2_(A-a)] didn’t differ between the two groups (P = 0.246). The differences of left ventricular ejection fraction (LVFS), left ventricular fractional shortening (LVFS) and D-dimer between the COPD patients with PH and without PH were not significant. In addition, there were significant differences in neutrophil count, albumin, creatinine, blood urea nitrogen (BUN) and cystatin C between the COPD patients with or without PH (all P < 0.05).

**Table 1:** Clinical and physiological characteristics of study population I

Characteristic	Total	COPD without pulmonary hypertension	COPD with pulmonary hypertension	P-value
Number	937	758	179	
Age (year)	65.00 (58.00–71.00)	65.00 (58.00–71.00)	68.00 (61.00–73.00)	0.000
Male (%)	77.6	75.7	85.5	0.005
Body mass index	23.63 ± 3.92	23.69 ± 3.87	22.34 ± 3.89	0.000
Smoking index (pack-year)	20.00 (0.00–40.00)	20.00 (0.00–40.00)	30.00 (9.00–40.00)	0.029
Smoking status				
Never (%)	33.3	35.8	22.9	
Former (%)	30.3	28.9	36.3	
Current (%)	36.4	35.4	40.8	
Comorbidity				
Hypertension (%)	28.1	27.7	29.6	0.610
Diabetes (%)	6.7	6.5	7.8	0.515
Coronary heart disease (%)	22.1	17.8	40.2	0.000
Inhalation therapy				
SABD (%)	2.7	2.4	3.9	0.955
ICS/LABA (%)	28.8	29.6	25.7	0.306
ICS/LABA + LAMA (%)	29.2	29.6	27.9	0.669
LAMA (%)	22.9	22.0	26.8	0.092
FEV_1_ (L)	1.14 (0.81–1.54)	1.18 (0.86–1.58)	0.87 (0.69–1.33)	0.000
FEV_1_ (% predicted)	45.60 (31.90–62.90)	48.00 (34.00–65.73)	34.90 (27.20–53.30)	0.000
FVC (L)	2.50 (2.00–3.06)	2.56 (2.03–3.12)	2.33 (1.90–2.70)	0.000
FVC (% predicted)	79.13 ± 21.22	80.74 ± 21.51	72.28 ± 18.53	0.000
FEV_1_/FVC (%)	46.37 (37.58–57.87)	47.54 (38.80–58.41)	41.08 (33.90–52.85)	0.000
VA (L)	4.91 ± 1.06	4.95 ± 1.07	4.79 ± 1.02	0.070
VA (% predicted)	86.55 ± 14.53	87.42 ± 14.31	82.85 ± 14.95	0.000
D_LCO_ (mmol/min/kPa)	5.81 ± 2.36	6.14 ± 2.32	4.42 ± 2.00	0.000
D_LCO_ (% predicted)	73.45 ± 27.80	77.25 ± 26.66	57.39 ± 26.87	0.000
FVC/D_LCO_ (l/mmol/min/kPa)	0.44 (0.35–0.56)	0.42 (0.34–0.53)	0.51 (0.41–0.76)	0.000
FVC%/D_LCO_%	1.06 (0.87–1.38)	1.03 (0.84–1.30)	1.26 (0.99–1.82)	0.000
LVEF (%)	67.00 (63.00–71.50)	67.00 (63.00–71.00)	67.00 (63.00–72.00)	0.427
LVFS (%)	37.00 (34.00–41.00)	37.00 (34.00–41.00)	37.00 (34.00–42.00)	0.539
pH	7.42 ± 0.03	7.43 ± 0.03	7.42 ± 0.04	0.002
PaO_2_ (mmHg)	70.80 (63.60–78.00)	71.60 (65.18–78.53)	66.50 (58.30–76.00)	0.000
PaCO_2_ (mmHg)	38.90 (35.60–42.70)	38.50 (35.20–42.00)	40.80 (36.60–47.90)	0.000
PO_2_(A-a) (mmHg)	27.10 (19.85–34.30)	26.90 (20.00–33.70)	28.00 (19.20–37.30)	0.246
Leukocyte count (× 10^9^/L)	6.42 (5.11–8.22)	6.38 (5.12–8.13)	6.71 (5.02–8.73)	0.369
Neutrophil count (× 10^9^/L)	4.21 (3.13–5.94)	4.13 (3.11–5.80)	4.49 (3.29–6.57)	0.024
Platelet count (× 10^9^/L)	181.00 (142.00–222.50)	183.00 (146.75–223.00)	172.00 (133.00–215.00)	0.054
Hemoglobin (g/L)	137.00 (125.00–146.00)	136.00 (125.00–146.00)	137.00 (125.00–148.00)	0.289
Albumin (g/L)	39.48 ± 3.99	39.66 ± 3.92	38.71 ± 4.19	0.004
Globulin (g/L)	24.79 ± 4.42	24.86 ± 4.30	24.53 ± 4.88	0.407
ALT (IU/L)	17.00 (12.00–25.00)	17.00 (12.00–25.25)	16.00 (12.00–24.00)	0.548
AST (IU/L)	19.00 (16.00–25.00)	19.00 (16.00–24.00)	20.00 (16.00–26.00)	0.060
DBIL (μmol/L)	4.41 (3.28–6.00)	4.40 (3.30–5.90)	4.70 (3.20–6.30)	0.261
IBIL (μmol/L)	6.97 (4.89–9.47)	6.90 (4.87–9.38)	7.10 (5.00–9.90)	0.143
Creatinine (μmol/L)	69.41 (59.33–81.00)	68.74 (58.80–80.33)	73.01 (62.00–83.00)	0.021
BUN (mmol/L)	5.08 (4.10–6.23)	4.99 (4.09–6.08)	5.45 (4.26–6.63)	0.009
Cystatin C (mg/L)	0.99 (0.86–1.13)	0.97 (0.85–1.11)	1.02 (0.88–1.20)	0.003
D-dimer (ng/mL)	360.00 (205.00–625.00)	340.00 (200.00–610.00)	430.00 (210.00–710.00)	0.052

Data are expressed as means ± standard deviation or median (interquartile range) or percentage

*SABD* short-acting bronchodilator, *ICS* inhaled corticosteroids, *LABA* long-acting beta-2 agonist, *LAMA* long-acting muscarinic antagonists, *FEV*_*1*_ forced expiratory volume in 1 s, *FVC* forced vital capacity, *VA* alveolar ventilation, *D*_*LCO*_ diffusing lung capacity for carbon monoxide, *LVEF* left ventricular ejection fraction, *LVFS* left ventricular fractional shortening, *PaO*_*2*_ partial pressure of oxygen in arterial blood, *PaCO*_*2*_ partial pressure of carbon dioxide in arterial blood, *PO2*_*(A-a)*_ alveolar-arterial oxygen gradient, *ALT* alanine aminotransferase, *AST* aspartate aminotransferase, *DBIL* direct bilirubin, *IBIL* indirect bilirubin, *BUN* blood urea nitrogen

**Fig. 2:**
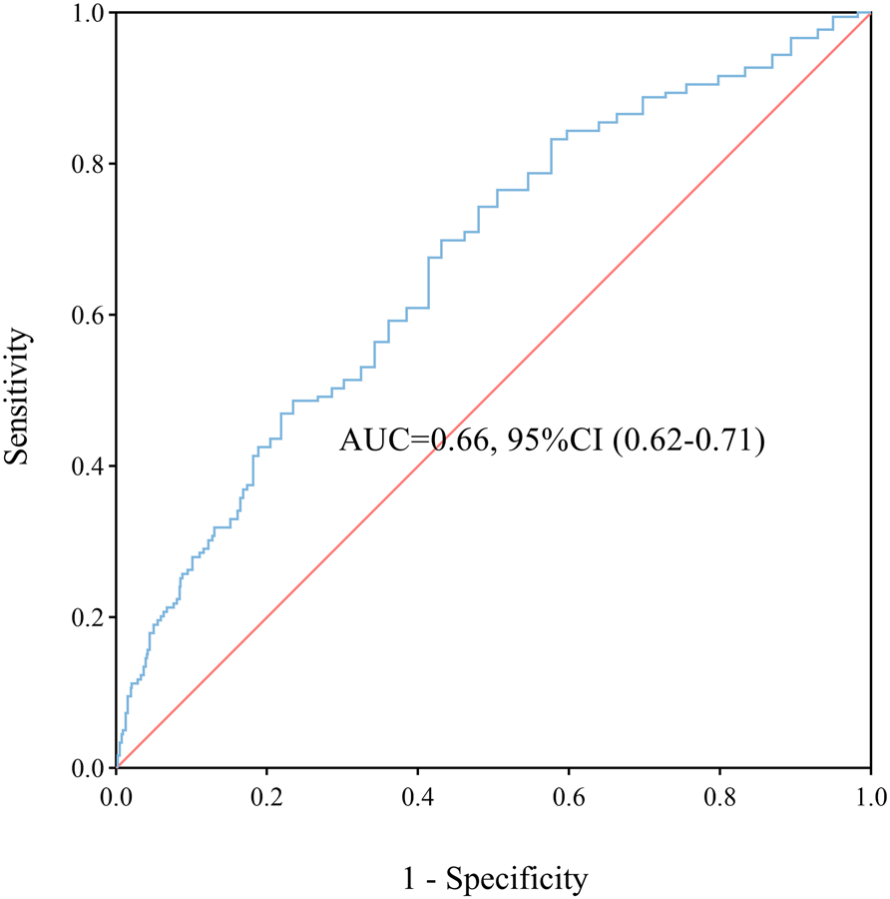
ROC curve for FVC/D_LCO_ as related to COPD with pulmonary hypertension in study population I. The cut-point of FVC/D_LCO_ value was 0.44 l/mmol/min/kPa. ROC: receiver operating characteristic

ROC was used to evaluate the diagnostic value of FVC/D_LCO_ on COPD with PH. As shown in Fig. 2, the area under the ROC curve (AUC) for FVC/D_LCO_ was 0.66 (95%CI 0.62–0.71, P < 0.001), and the threshold for FVC/D_LCO_ was 0.44 l/mmol/min/kPa. The study population I was further divided into two groups according to the FVC/D_LCO_ threshold, and the clinical and physiological characteristics are shown in the supplementary data (Additional file 1: Table S1). When the value of FVC/D_LCO_ was greater than 0.44 l/mmol/min/kPa in patients with COPD, these patients were more likely to combine with PH (26.1% vs. 12.1%, P < 0.001). Moreover, there were significant differences in FEV_1_/FVC, VA, D_LCO_, PO_2_(A-a), platelet count, albumin, aspartate aminotransferase, indirect bilirubin, creatinine, BUN and cystatin C when the study population I was stratified by FVC/D_LCO_ threshold (all P < 0.05).

### Factors associated with COPD combined with PH

Based on published literatures and relevant expertise, demographic information, comorbidities, partial data of pulmonary function and blood gas analysis, as well as the laboratory parameters with statistical differences were brought into logistic regression analysis. And the results of univariate and multivariate associations with PH in COPD patients are presented in Table 2. Univariate logistic regression analysis revealed that older age, D_LCO_% predicted < 80%, FVC/D_LCO_ ≥ 0.44 l/mmol/min/kPa, coexistence with coronary heart disease, PaO_2_ < 60 mmHg, PaCO_2_ ≥ 50 mmHg, and elevated levels of neutrophil count, BUN and cystatin C all significantly increased the odds of combining PH in COPD patients. And there was no significant correlation between inhalation therapy and the risk of COPD combined with PH. After controlling the relevant covariates, FVC/D_LCO_ ≥ 0.44 l/mmol/min/kPa remained a strong predictor for PH in patients with COPD (OR = 2.11, 95%CI 1.30–3.45, P = 0.003).

**Table 2:** Univariate and multivariate associations with pulmonary hypertension in COPD

Variable	Univariate Analysis		Multivariate Analysis	
	OR (95% CI)	P-value	OR (95% CI)	P-value
Age (per increase of 1-year)	1.04 (1.02–1.06)	0.000	1.03 (1.00–1.06)	0.021
Sex (female vs male)	0.53 (0.34–0.83)	0.005	0.89 (0.52–1.53)	0.676
Body mass index (per increase of 1 point)	0.91 (0.87–0.95)	0.000	0.94 (0.89–0.99)	0.019
Smoking index (pack-year)	1.00 (0.99–1.01)	0.202		
FEV_1_ (% predicted)	0.97 (0.96–0.98)	0.000	0.98 (0.97–1.00)	0.080
FVC (% predicted)	0.98 (0.97–0.99)	0.000	0.99 (0.98–1.01)	0.647
VA (% predicted)	0.97 (0.96–0.98)	0.000	1.01 (0.99–1.02)	0.528
D_LCO_ (% predicted) (≥ 80 vs < 80%)	3.14 (2.13–4.63)	0.000	1.03 (0.59–1.80)	0.911
FVC/D_LCO_ (≥ 0.44 vs < 0.44 l/mmol/min/kPa)	2.56 (1.18–3.62)	0.000	2.11 (1.30–3.45)	0.003
Hypertension (yes vs no)	1.10 (0.77–1.57)	0.610		
Diabetes (yes vs no)	1.23 (0.66–2.28)	0.515		
Coronary heart disease (yes vs no)	3.11 (2.18–4.12)	0.000	3.23 (2.15–4.84)	0.000
SABD (yes vs no)	1.18 (0.43–3.23)	0.745		
ICS/LABA (yes vs no)	0.83 (0.57–1.19)	0.306		
ICS/LABA + LAMA (yes vs no)	0.92 (0.64–1.33)	0.669		
LAMA (yes vs no)	1.37 (0.95–1.98)	0.093		
pH (≥ 7.4 vs < 7.4)	0.54 (0.37–0.78)	0.001	0.91 (0.57–1.45)	0.701
PaO_2_ (≥ 60 vs < 60 mmHg)	3.47 (2.36–5.10)	0.000	1.76 (1.10–2.82)	0.019
PaCO_2_ (≥ 50 vs < 50 mmHg)	4.50 (2.70–7.50)	0.000	3.13 (1.57–6.23)	0.001
Neutrophil count (per increase of 1 × 10^9^/L)	1.11 (1.04–1.18)	0.002	1.07 (0.99–1.15)	0.063
Albumin (per increase of 1 standard deviation g/L)	0.94 (0.91–0.98)	0.005	1.01 (0.96–1.06)	0.726
Creatinine (per increase of 1 standard deviation μmol/L)	1.01 (1.00–1.02)	0.052		
BUN (per increase of 1 standard deviation mmol/L)	1.15 (1.06–1.26)	0.001	1.04 (0.94–1.15)	0.480
Cystatin C (per increase of 1standard deviation mg/L)	3.03 (1.64–5.61)	0.000	1.97 (0.94–4.13)	0.073

*OR* odds ratio, *95% CI* 95% confidence interval, *FEV*_*1*_ forced expiratory volume in 1 s, *FVC* forced vital capacity, *VA* alveolar ventilation, *D*_*LCO*_ diffusing lung capacity for carbon monoxide, *SABD* short-acting bronchodilator, *ICS* inhaled corticosteroids, *LABA* long-acting beta-2 agonist, *LAMA* long-acting muscarinic antagonists, *PaO*_*2*_ partial pressure of oxygen in arterial blood, *PaCO*_*2*_ partial pressure of carbon dioxide in arterial blood, *BUN* blood urea nitrogen

### Characteristics of the patients in the study population II

In order to explore the 5-year all-cause mortality of COPD patients in the study population I, a total of 750 patients were available to the telephone follow-up (Study population II), and the clinical physiological characteristics of the study population II are shown in Table 3. Our present study displayed that the 5-year all-cause mortality of COPD patients was significantly higher in combined with PH group than without PH group (32.0% vs 13.0%, P < 0.001). Non-survivors were older at a median age of 68.50 years, and had a lower BMI and a higher smoking index than survivors (P < 0.05). The proportion of PH in the study population II was higher in non-survivor group than that in the survivor group (33.6% vs. 13.9%, P < 0.001). And there were no significant differences in the inhalation therapy between the non-survivor group and survivor group. Non-survivors had severe airflow obstruction, and moderate diffusing capacity impairment and a higher FVC/D_LCO_ value compared with the survivors (P < 0.001). And VA was significantly decreased in non-survivor group compared to the survivor group (P < 0.01). PaO_2_ was decreased, and PaCO_2_ as well as PO_2_(A-a) was increased in the non-survivor group compared with the survivor group (P < 0.05). In addition, the levels of neutrophil count, cystatin C and D-dimer all significantly increased, and alanine aminotransferase concentration notably decreased in the non-survivor group (P < 0.05). And there were no significant differences in LVEF, LVFS, leukocyte count, platelet count, hemoglobin, globulin, aspartate aminotransferase, direct bilirubin, indirect bilirubin, creatinine and BUN between non-survivor and survivor groups.

**Table 3:** Clinical and physiological characteristics of study population II

Characteristic	Total	Non-survivors	Survivors	P-value
Number	750	122	628	
Age (year)	65.00 (58.00–71.00)	68.50 (61.75–74.00)	64.00 (58.00–71.00)	0.000
Male (%)	77.2	82.8	76.1	0.108
Body mass index	23.63 ± 3.92	21.93 ± 3.85	23.96 ± 3.85	0.000
Smoking index (pack-yr.)	20.00 (0.00–40.00)	30.00 (0.00–45.00)	20.00 (0.00–40.00)	0.033
Smoking status				
Never (%)	33.1	26.2	34.4	
Former (%)	30.5	34.4	29.8	
Current (%)	36.4	39.3	35.8	
Comorbidity				
Hypertension (%)	28.8	36.1	27.4	0.053
Diabetes (%)	6.9	7.4	6.8	0.833
Coronary heart disease (%)	21.6	28.7	20.2	0.038
Pulmonary hypertension (%)	17.1	33.6	13.9	0.000
Inhalation therapy				
SABD (%)	2.7	4.1	2.4	0.444
ICS/LABA (%)	28.1	25.4	28.7	0.465
ICS/LABA + LAMA (%)	28.8	22.1	30.1	0.075
LAMA (%)	25.5	32.0	24.2	0.072
FEV_1_ (L)	1.16 (0.83–1.55)	0.88 (0.71–1.21)	1.22 (0.87–1.62)	0.000
FEV_1_ (% predicted)	46.50 (32.48–63.45)	34.35 (27.10–49.98)	48.65 (35.15–66.18)	0.000
FVC (L)	2.51 (2.03–3.08)	2.27 (1.80–2.65)	2.59 (2.09–3.16)	0.000
FVC (% predicted)	79.96 ± 21.11	72.42 ± 20.28	81.43 ± 20.97	0.000
FEV_1_/FVC (%)	46.99 (37.93–58.15)	41.19 (32.83–53.72)	48.48 (39.10–58.50)	0.000
VA (L)	4.93 ± 1.07	4.68 ± 0.99	4.98 ± 1.08	0.004
VA (% predicted)	87.02 ± 14.55	82.20 ± 15.06	87.95 ± 14.27	0.000
D_LCO_ (mmol/min/kPa)	5.89 (4.41–7.39)	4.12 (3.24–5.45)	6.18 (4.74–7.70)	0.000
D_LCO_ (% predicted)	75.41 ± 27.54	56.16 ± 24.53	79.15 ± 26.53	0.000
FVC/D_LCO_ (l/mmol/min/kPa)	0.42 (0.34–0.54)	0.52 (0.41–0.71)	0.41 (0.33–0.52)	0.000
FVC%/D_LCO_%	1.04 (0.85–1.33)	1.26 (1.00–1.65)	1.01 (0.83–1.26)	0.000
LVEF (%)	67.00 (62.75–71.00)	66.00 (61.00–71.25)	67.00 (63.00–71.00)	0.104
LVFS (%)	37.00 (34.00–41.00)	37.00 (33.00–42.00)	37.00 (34.00–41.00)	0.789
pH	7.43 ± 0.03	7.42 ± 0.04	7.43 ± 0.03	0.619
PaO_2_ (mmHg)	71.56 ± 13.29	66.48 ± 14.86	72.55 ± 12.74	0.000
PaCO_2_ (mmHg)	38.80 (35.58–42.40)	39.95 (36.25–46.58)	38.55 (35.40–41.70)	0.001
PO_2_(A-a) (mmHg)	26.90 (19.70–33.83)	27.70 (20.85–38.70)	26.70 (19.60–33.60)	0.031
Leukocyte count (× 10^9^/L)	6.51 (5.18–8.25)	6.66 (5.32–8.21)	6.45 (5.16–8.27)	0.650
Neutrophil count (× 10^9^/L)	4.22 (3.16–5.98)	4.52 (3.42–6.39)	4.16 (3.10–5.84)	0.039
Platelet count (× 10^9^/L)	179.00 (141.00–226.00)	166.50 (135.50–210.50)	182.00 (143.25–227.00)	0.083
Hemoglobin (g/L)	135.86 ± 16.96	133.66 ± 19.93	136.29 ± 16.30	0.118
Albumin (g/L)	39.80 (37.00–42.40)	38.05 (35.60–40.80)	40.00 (37.30–42.60)	0.000
Globulin (g/L)	24.84 ± 4.39	25.25 ± 4.96	24.75 ± 4.27	0.305
ALT (IU/L)	17.00 (12.00–25.00)	15.08 (11.00–22.00)	17.00 (12.00–26.00)	0.046
AST (IU/L)	19.00 (16.00–25.00)	20.00 (15.00–26.00)	19.00 (16.00–24.96)	0.663
DBIL (μmol/L)	4.50 (3.36–6.00)	4.65 (3.25–6.44)	4.42 (3.40–5.91)	0.295
IBIL (μmol/L)	6.87 (4.79–9.43)	6.62 (4.57–9.84)	6.90 (4.88–9.40)	0.587
Creatinine (μmol/L)	68.93 (58.67–80.33)	69.13 (59.37–81.77)	68.72 (58.67–80.28)	0.624
BUN (mmol/L)	5.10 (4.11–6.19)	5.24 (4.28–6.36)	5.08 (4.05–6.12)	0.135
Cystatin C (mg/L)	0.99 (0.86–1.13)	1.04 (0.89–1.21)	0.98 (0.85–1.11)	0.007
D-dimer (ng/mL)	360.00 (210.00–620.00)	461.50 (270.00–742.50)	330.00 (210.00–600.00)	0.002

Data are expressed as means ± standard deviation or median (interquartile range) or percentage

*SABD* short-acting bronchodilator, *ICS* inhaled corticosteroids, *LABA* long-acting beta-2 agonist, *LAMA* long-acting muscarinic antagonists, *FEV*_*1*_ forced expiratory volume in 1 s, *FVC* forced vital capacity, *VA* alveolar ventilation, *D*_*LCO*_ diffusing lung capacity for carbon monoxide, *LVEF* left ventricular ejection fraction, *LVFS* left ventricular fractional shortening, *PaO*_*2*_, partial pressure of oxygen in arterial blood, *PaCO*_*2*_ partial pressure of carbon dioxide in arterial blood, *PO2*_*(A-a)*_ alveolar-arterial oxygen gradient, *ALT* alanine aminotransferase, *AST* aspartate aminotransferase, *DBIL* direct bilirubin, *IBIL* indirect bilirubin, *BUN* blood urea nitrogen

**Fig. 3:**
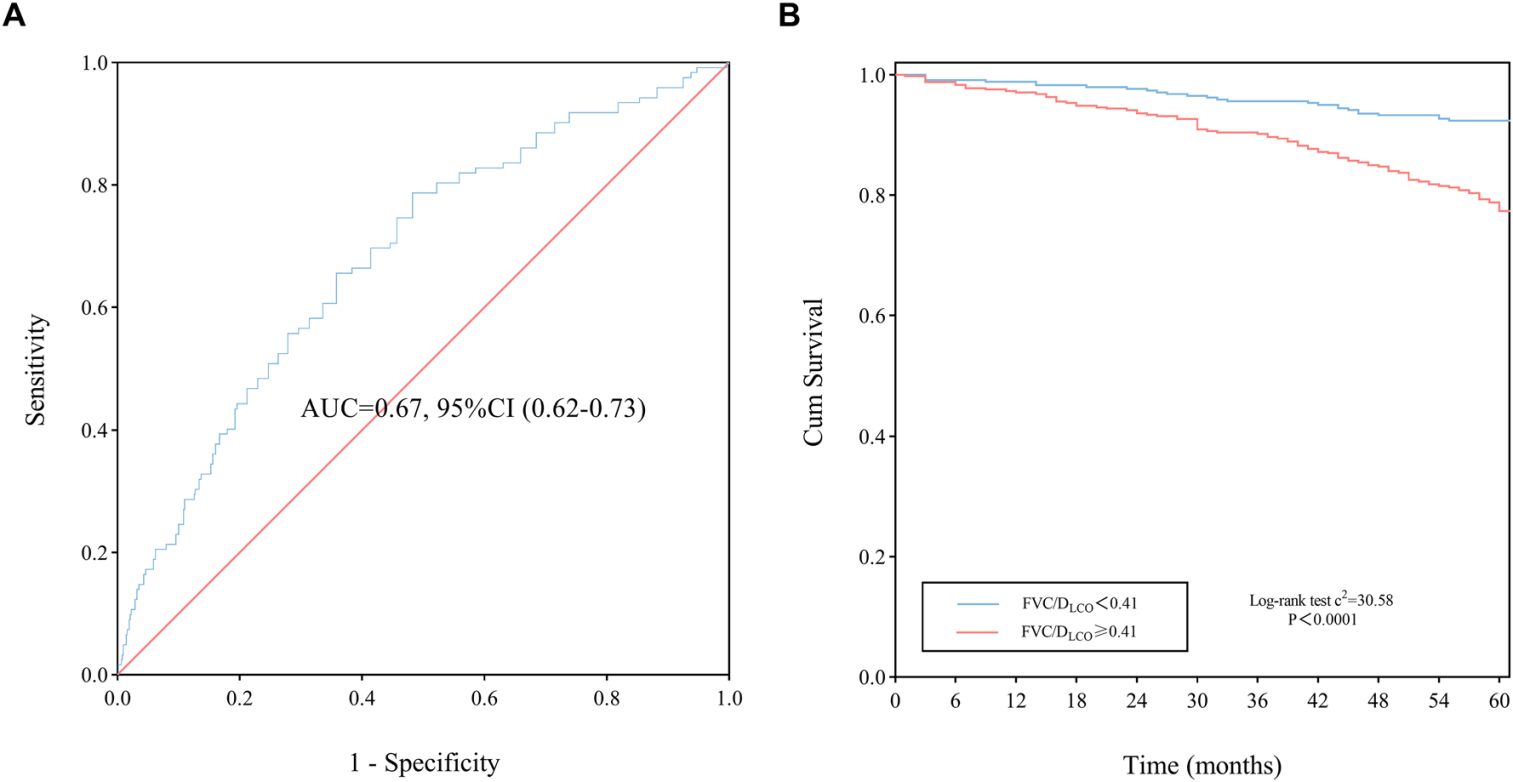
ROC curve for FVC/D_LCO_ and survival curves of study population II. **A** ROC curve for FVC/D_LCO_ as related to 5-year all-cause mortality of COPD patients. The cut-point of FVC/D_LCO_ value was 0.41 l/mmol/min/kPa. **B** Kaplan–Meier survival curves of COPD patients according to the cut-point of FVC/D_LCO_. Red line refers to FVC/D_LCO_ ≥ 0.41 l/mmol/min/kPa, and blue line refers to FVC/D_LCO_ < 0.41 l/mmol/min/kPa. *ROC* receiver operating characteristic

ROC was used to evaluate the diagnostic value of FVC/D_LCO_ on 5-year all-cause mortality of COPD patients. The AUC for FVC/D_LCO_ was 0.67 (95%CI 0.62–0.73, P < 0.001), and the threshold for FVC/D_LCO_ was 0.41 l/mmol/min/kPa which were shown in Fig. 3A. And Kaplan–Meier survival curves showed that 5-year cumulative survival rate for COPD patients were decreased when the value of FVC/D_LCO_ was ≥ 0.41 l/mmol/min/kPa (log-rank test c^2^ = 30.58, P < 0.0001, Fig. 3B). According to the threshold of FVC/D_LCO_, the study population II were further divided into two groups, and the clinical and physiological characteristics are shown in the supplementary data (Additional file 1: Table S2). The 5-year all-cause mortality and PH incidence were significantly increased when the value of FVC/D_LCO_ was ≥ 0.41 l/mmol/min/kPa in patients with COPD (23.5% vs. 7.6%, and 22.5% vs. 10.6% respectively, both P < 0.001). Moreover, there were significant differences in BMI, smoking index, FVC, FEV_1_/FVC, VA, D_LCO_, platelet count, hemoglobin, albumin, aspartate aminotransferase, creatinine and cystatin C when the study population II was classified by FVC/D_LCO_ threshold for COPD mortality (all P < 0.05).

### Factors associated with 5-year all-cause mortality in COPD patients

The univariate and multivariate associations with 5-year all-cause mortality in patients with COPD as shown in Table 4. In the univariate cox regression analysis, FVC/D_LCO_ was an independent predictor of 5-year all-cause mortality in COPD patients (HR = 3.33, 95%CI 2.16–5.13, P < 0.001), along with age, BMI, FEV_1_%, FVC%, VA%, D_LCO_%, the comorbidity (hypertension, coronary heart disease or PH), pH, PaO_2_, PaCO_2_, neutrophil count, albumin and cystatin C. And the inhalation therapy had no significant effect on the 5-year all-cause mortality of COPD patients. The multivariate cox regression analysis showed that FVC/D_LCO_ was a significant predictor for 5-year all-cause mortality of COPD patients (HR = 2.05, 95%CI 1.19–3.53, P = 0.009). In addition, age, BMI, comorbidity (hypertension or PH), PaCO_2_, and albumin were also significantly correlated with COPD prognosis (all P < 0.05).

**Table 4:** Univariate and multivariate associations with 5-year all-cause mortality of COPD

Variable	Univariate Analysis		Multivariate Analysis	
	HR (95%CI)	P-value	HR (95%CI)	P-value
Age (per increase of 1-year)	1.05 (1.02–1.07)	0.000	1.04 (1.01–1.07)	0.007
Sex (female vs male)	0.69 (0.43–1.10)	0.121		
Body mass index (per increase of 1 point)	0.88 (0.84–0.92)	0.000	0.93 (0.88–0.98)	0.011
Smoking index (pack-year)	1.00 (0.99–1.01)	0.077		
FEV_1_ (% predicted)	0.97 (0.96–0.98)	0.000	0.99 (0.97–1.01)	0.268
FVC (% predicted)	0.98 (0.97–0.99)	0.000	1.00 (0.98–1.02)	0.712
VA (% predicted)	0.97(0.96–0.99)	0.000	1.00 (0.98–1.01)	0.518
D_LCO_ (% predicted) (≥ 80 vs < 80%)	4.50 (2.76–7.34)	0.000	1.37 (0.73–2.58)	0.323
FVC/D_LCO_ (≥ 0.41 vs < 0.41 l/mmol/min/kPa)	3.33 (2.16–5.13)	0.000	2.05 (1.19–3.53)	0.009
Hypertension (yes vs no)	1.47 (1.02–2.24)	0.041	1.65 (1.09–2.49)	0.017
Diabetes (yes vs no)	1.07 (0.54–2.11)	0.840		
Coronary heart disease (yes vs no)	1.51 (1.02–2.24)	0.038	1.13 (0.74–1.73)	0.572
Pulmonary hypertension (yes vs no)	2.87 (1.97–4.17)	0.000	1.68 (1.12–2.53)	0.012
SABD (yes vs no)	1.70 (0.70–4.16)	0.245		
ICS/LABA (yes vs no)	0.86 (0.57–1.29)	0.461		
ICS/LABA + LAMA (yes vs no)	0.69 (0.45–1.05)	0.083		
LAMA (yes vs no)	1.40 (0.95–2.04)	0.086		
pH (≥ 7.4 vs < 7.4)	0.62 (0.42–0.93)	0.020	0.91 (0.57–1.45)	0.686
PaO_2_ (≥ 60 vs < 60 mmHg)	2.76 (1.88–4.06)	0.000	1.27 (0.81–2.00)	0.306
PaCO_2_ (≥ 50 vs < 50 mmHg)	3.34 (2.04–5.45)	0.000	2.02 (1.08–3.78)	0.028
Neutrophil count (per increase of 1 × 10^9^/L)	1.09 (1.02–1.16)	0.014	1.03 (0.97–1.09)	0.404
Albumin (per increase of 1 standard deviation g/L)	0.89 (0.85–0.93)	0.000	0.93 (0.89–0.97)	0.002
ALT (per increase of 1 standard deviation IU/L)	1.00 (0.99–1.01)	0.983		
Cystatin C (per increase of 1standard deviation mg/L)	2.36 (1.30–4.29)	0.005	1.22 (0.62–2.38)	0.569
D-dimer (per increase of 1standard deviation ng/mL)	1.00 (1.00–1.00)	0.476		

*HR* relative risk, *95% CI* 95% confidence interval, *FEV*_*1*_ forced expiratory volume in 1 s, *FVC* forced vital capacity, *VA* alveolar ventilation, *D*_*LCO*_ diffusing lung capacity for carbon monoxide, *SABD* short-acting bronchodilator, *ICS* inhaled corticosteroids, *LABA* long-acting beta-2 agonist, *LAMA* long-acting muscarinic antagonists, *PaO*_*2*_ partial pressure of oxygen in arterial blood, *PaCO*_*2*_ partial pressure of carbon dioxide in arterial blood, *ALT* alanine aminotransferase

## Discussion

Our present study found that PH incidence and 5-year all-cause mortality in COPD patients were significantly increased when the value of FVC/D_LCO_ was ≥ 0.44 and 0.41 l/mmol/min/kPa respectively. Multivariate regression analysis showed that FVC/D_LCO_ was a strong predictor for PH incidence and 5-year all-cause mortality in patients with COPD.

It has been reported that the presence and severity of PH was strongly associated with the prognosis of COPD [28]. As early as 1981, it has been proved that the 7-year survival rate of COPD patients with PH was 29.2% compared to 55.6% for COPD without PH [29]. The results from the ASPIRE Registry showed that 1-year and 3-year survival for severe PH were 70% and 33%, which was inferior to 83% and 55% respectively for mild-moderate PH in patients with COPD [30]. Our present study also demonstrated that the risk of death increased by 68% in COPD patients combined with PH, and the 5-year survival rate of COPD combined with PH was 68% compared to 87% in the patients without PH. It is very important to recognize PH in COPD patients, however PH is usually detected late in the course of COPD, with a majority of patients displaying severe functional compromise. A French national prospective study showed that PH was diagnosed approximately 27 months after the onset of the clinical symptoms [31]. The results of the REVEAL Registry revealed that 21.1% of patients experiences more than 2 years delay from the clinical symptom occurrence to the diagnosis of COPD [32]. Therefore, we should actively look for tools or methods that facilitate early identification PH in the patients with COPD.

The loss of FVC in the patients with COPD may be caused by hyperinflation or air trapping [33]. It has also been proved that the presence of PH further decreases lung diffusion function rather than maldistribution of ventilation in COPD [34], which is associated with the impaired pulmonary capillary bed. Therefore, we hypothesized that FVC/D_LCO_ could be used clinically to identify PH in COPD patients due to inconsistent decline of FVC and D_LCO_. Our present data indicates that FVC/D_LCO_ value ≥ 0.44 l/mmol/min/kPa could be used as a predictor of identifying PH in COPD patients, and 26.1% of COPD patients combined with PH when FVC/D_LCO_ was ≥ 0.44 l/mmol/min/kPa. The multivariate logistic regression analysis showed that hypoxemia and hypercapnia also were the risk factors for PH in COPD patients, which is consistent with previous study [12]. COPD is typically characterized by irreversible airflow limitation, and the decline of lung function induces hypoxemia and hypercapnia, which results in the development of PH in COPD patients. In addition, our results also suggested that the risk of combined PH in COPD patients was increased by 2.23 times when combined with coronary artery disease. Although coronary artery disease usually does not directly cause PH, the underlying mechanism may include increased oxygen consumption and severe chronic left heart failure.

Our present study has demonstrated that FVC/D_LCO_ is an important parameter to recognize PH in COPD patients, but it is unknown whether FVC/D_LCO_ is a meaningful factor to predict the prognosis of COPD. The values of FVC and D_LCO_ gradually decrease with the progression of COPD, but D_LCO_ decreases at a faster rate [35]. Thus, we hypothesized that an increase in the value of FVC/D_LCO_ could reflect the severity of COPD. Our present study displayed that the 5-year all-cause mortality of COPD patients was 23.5% when FVC/D_LCO_ was ≥ 0.41 l/mmol/min/kPa, compared to 7.6% in FVC/D_LCO_ < 0.41 l/mmol/min/kPa group. Further multivariate cox regression analysis showed that FVC/D_LCO_ was an independent predictor for 5-year all-cause mortality of COPD patients rather than FVC%/DLCO%, although FVC%/DLCO% values are related to mean pulmonary artery pressure in subjects with suspected PH [24]. And patients with FVC/D_LCO_ ≥ 0.41 l/mmol/min/kPa had 2.05 times death risk compared to FVC/D_LCO_ < 0.41 l/mmol/min/kPa.

It has been proven that long-term inhalation therapy can improve the prognosis of COPD [36]. However, the univariate cox regression analysis showed that there was no significant effect of inhalation therapy on COPD prognosis in our present study. The inconsistent results may be related to the following reasons. First, our study is a retrospective cohort study, and we did not have a regular follow-up from 2010 to 2017. Thus, we can only obtain the inhalation therapy status when COPD patients left the hospital, and this information may not reflect the true prognosis for COPD. Second, many patients alternately used SABD, ICS/LABA, LAMA or ICS/LABA + LAMA, and some patients even didn’t adhere to long-term inhalation therapy. So the irregular use of inhalation therapy may contribute to our present results. In addition, it has been reported that the degree of decline in D_LCO_ is strongly related with COPD prognosis, and a D_LCO_% < 60% predicted is associated with increased death risk and worse clinical presentation in the COPD patients with GOLD stage I [37]. However, our present study showed that D_LCO_% < 80% was not significant in predicting the 5-year all-cause mortality of COPD patients, which may be related to the fact that 80% predicted is the lower limit of the normal value for D_LCO_. Although the result of study population I indicated that PaO_2_ less than 60 mmHg was significant for identifying PH in COPD patients, it is not an independent risk factor for 5-year all-cause death, which may be due to the fact that some patients in the study population II had received standardized treatment including oxygen therapy. Therefore, we can use FVC/D_LCO_ to stratify the high death risk of COPD patients and pay more attention to these patients.

There are several limitations that should be mentioned. First, medical treatments including regular long-term inhalation therapy may be potential confounding factors for assessing the role of FVC/D_LCO_ in COPD. In order to exclude the confounding effects of medical treatments on our present results, a prospective cohort study with regular follow-up should be carried out in the future. Second, there may be some errors in PH diagnosis according to echocardiography. At the same time, we cannot study the relation between pulmonary artery pressure value and FVC/D_LCO_ due to the incomplete data of pulmonary artery pressure in COPD patients. In the future studies, we should determine and record the pulmonary artery pressure value by echocardiography or RHC, and further explore its relation with FVC/D_LCO_ in COPD. Third, the specific death cause of COPD patients was not recorded in our study, thus the factors influencing the death of COPD could not be further explored. Finally, we can further clarify whether one cut-off value of FVC/D_LCO_ can be used to predict PH incidence in COPD and the 5-year all-cause mortality of COPD through a larger multicenter cohort study.

## Conclusion

In conclusion, our study has shown that FVC/D_LCO_ can not only be used to identify PH in COPD patients, but also is an independent predictor for 5-year all-cause mortality in COPD patients. This non-invasive evaluation tool may provide useful value for the patients with COPD.

## Supplementary Information

**Additional file 1: Table S1.** Clinical and physiological characteristics of study population I stratified by FVC/DLCO. Table S2. Clinical and physiological characteristics of study population II classified by FVC/DLCO.

## Data Availability

The datasets and analysis of this study are available from the corresponding author on reasonable request.
