# Supplementary material for: FVC/DLCO identifies pulmonary hypertension and predicts 5-year all-cause mortality in patients with COPD
Source: Eur J Med Res. 2023 May 15;28:174. doi: 10.1186/s40001-023-01130-6 (PMC10184375; doi:10.1186/s40001-023-01130-6)
Supplement: Supplementary file 1 — Additional file 1: Table S1. Clinical and physiological characteristics of study population I stratified by FVC/DLCO. Table S2. Clinical and physiological characteristics of study population II classified by FVC/DLCO. [file 40001_2023_1130_MOESM1_ESM.docx]

**Table S1** Clinical and physiological characteristics of study population Ⅰ stratified by FVC/D_LCO_

| **Characteristic** | **FVC/D_LCO_≥0.44 l/mmol/min/kPa** | **FVC/D_LCO_＜0.44 l/mmol/min/kPa** | **P-value** |
| --- | --- | --- | --- |
| Number | 467 | 470 |  |
| Age (yr.) | 67.00 (60.00-72.00) | 64.00 (57.00-70.00) | .000 |
| Male (%) | 86.5 | 68.7 | .000 |
| Body mass index | 22.34±3.54 | 24.51±3.97 | .000 |
| Smoking index (pack-yr.) | 30.00 (7.50-40.00) | 11.25 (0.00-40.00) | .000 |
| Smoking status |  |  |  |
| Never (%) | 23.1 | 43.4 |  |
| Former (%) | 34.7 | 26.0 |  |
| Current (%) | 42.2 | 30.6 |  |
| Comorbidity |  |  |  |
| Hypertension (%) | 27.2 | 28.9 | .553 |
| Diabetes (%) | 5.1 | 8.3 | .054 |
| Coronary heart disease (%) | 25.1 | 19.1 | .029 |
| Pulmonary hypertension (%) | 26.1 | 12.1 | .000 |
| FEV_1_ (L) | 1.18 (0.81-1.58) | 1.11 (0.82-1.49) | .081 |
| FEV_1_ (% predicted) | 45.00 (31.10-63.30) | 46.00 (32.40-62.20) | .948 |
| FVC (L) | 2.65 (2.20-3.23) | 2.35 (1.90-2.89) | .000 |
| FVC (% predicted) | 81.35±22.37 | 76.92±19.80 | .001 |
| FEV_1_/FVC (%) | 45.06 (35.82-56.18) | 49.36 (39.03-59.78) | .000 |
| VA (L) | 5.06±1.04 | 4.77±1.07 | .000 |
| VA (% predicted) | 85.95±14.55 | 87.14±14.51 | .209 |
| D_LCO_ (mmol/min/kPa) | 4.52±1.93 | 7.10±2.02 | .000 |
| D_LCO_ (% predicted) | 56.31±22.35 | 90.49±21.52 | .000 |
| FVC/D_LCO_ (l/mmol/min/kPa) | 0.56 (0.49-0.74) | 0.35 (0.31-0.39) | .000 |
| FVC%/D_LCO_% | 1.38(1.16-1.76) | 0.87(0.75-0.97) | .000 |
| LVEF (%) | 67.00 (63.00-72.00) | 67.00 (62.00-71.00) | .102 |
| LVFS (%) | 37.00 (34.00-41.00) | 37.00 (33.00-41.00) | .159 |
| pH | 7.43±0.03 | 7.42±0.03 | .021 |
| PaO_2_ (mmHg) | 70.50 (62.30-78.00) | 71.15 (65.10-77.85) | .152 |
| PaCO_2_ (mmHg) | 38.50 (35.10-42.80) | 39.00 (36.10-42.63) | .050 |
| PO_2_(A-a) (mmHg) | 27.70 (20.40-35.70) | 26.20 (19.30-33.00) | .015 |
| Leukocyte count (×10^9^/L) | 6.37 (5.10-7.90) | 6.53 (5.11-8.37) | .484 |
| Neutrophil count (×10^9^/L) | 4.24 (3.20-5.97) | 4.18 (3.01-5.94) | .559 |
| Platelet count (×10^9^/L) | 173.00 (135.00-209.00) | 187.00 (152.75-231.00) | .000 |
| Hemoglobin (g/L) | 137.00 (125.00-146.00) | 136.50 (127.00-147.00) | .468 |
| Albumin (g/L) | 38.88±4.01 | 40.07±3.89 | .000 |
| Globulin (g/L) | 24.70±4.57 | 24.89±4.25 | .529 |
| ALT (IU/L) | 17.00 (12.00-25.00) | 16.00 (12.00-25.00) | .948 |
| AST (IU/L) | 20.00 (17.00-26.00) | 19.00 (15.00-24.00) | .001 |
| DBIL (μmol/L) | 4.53 (3.28-6.18) | 4.37 (3.27-5.79) | .136 |
| IBIL (μmol/L) | 7.23 (5.00-9.90) | 6.82 (4.62-9.10) | .024 |
| Creatinine (μmol/L) | 72.30 (62.40-83.44) | 67.05 (56.33-78.00) | .000 |
| BUN (mmol/L) | 5.16 (4.21-6.33) | 4.95 (4.01-6.07) | .041 |
| Cystatin C (mg/L) | 1.00 (0.87-1.16) | 0.97 (0.84-1.11) | .005 |
| D-dimer (ng/mL) | 360.00 (188.00-640.00) | 350.00 (220.00-610.00) | .590 |

**Notes:** Data are expressed as means ± standard deviation or median (interquartile range) or percentage.

**Abbreviations:** FEV_1_, forced expiratory volume in 1 second; FVC, forced vital capacity; VA, alveolar ventilation; D_LCO_, diffusing lung capacity for carbon monoxide; LVEF, left ventricular ejection fraction; LVFS, left ventricular fractional shortening; PaO_2_, partial pressure of oxygen in arterial blood; PaCO_2_, partial pressure of carbon dioxide in arterial blood; PO2_(A-a)_, alveolar-arterial oxygen gradient; ALT, alanine aminotransferase; AST, aspartate aminotransferase; DBIL, direct bilirubin; IBIL, indirect bilirubin; BUN, blood urea nitrogen.

**Table S2** Clinical and physiological characteristics of study population Ⅱ classified by FVC/D_LCO_

| **Characteristic** | **FVC/D_LCO_≥0.41 l/mmol/min/kPa** | **FVC/D_LCO_＜0.41 l/mmol/min/kPa** | **P-value** |
| --- | --- | --- | --- |
| Number | 409 | 341 |  |
| Age (yr.) | 66.00 (60.00-72.00) | 63.00 (57.00-70.00) | .000 |
| Male (%) | 86.6 | 66 | .000 |
| Body mass index | 22.44±3.57 | 25.07±3.85 | .000 |
| Smoking index (pack-yr.) | 30.00 (6.75-40.00) | 10.00 (0.00-38.75) | .000 |
| Smoking status |  |  |  |
| Never (%) | 22.7 | 45.5 |  |
| Former (%) | 36.2 | 23.8 |  |
| Current (%) | 41.1 | 30.8 |  |
| Comorbidity |  |  |  |
| Hypertension (%) | 26.7 | 31.4 | .155 |
| Diabetes (%) | 4.6 | 9.7 | .007 |
| Coronary heart disease (%) | 24.0 | 18.8 | .085 |
| Pulmonary hypertension (%) | 22.5 | 10.6 | .000 |
| FEV_1_ (L) | 1.19 (0.84-1.60) | 1.13 (0.83-1.50) | .053 |
| FEV_1_ (% predicted) | 45.90 (32.00-63.50) | 46.80 (32.85-63.50) | .890 |
| FVC (L) | 2.69 (2.23-3.25) | 2.35 (1.85-2.90) | .000 |
| FVC (% predicted) | 82.26±21.62 | 77.21±20.16 | .001 |
| FEV_1_/FVC (%) | 45.23 (36.24-56.57) | 50.84 (40.33-60.14) | .000 |
| VA (L) | 5.09±1.02 | 4.75±1.10 | .000 |
| VA (% predicted) | 86.63±14.36 | 87.48±14.77 | .429 |
| D_LCO_ (mmol/min/kPa) | 4.74 (3.54-6.06) | 7.00 (5.91-8.60) | .000 |
| D_LCO_ (% predicted) | 60.34±22.38 | 93.50±21.65 | .000 |
| FVC/D_LCO_ (l/mmol/min/kPa) | 0.53 (0.46-0.71) | 0.33 (0.30-0.38) | .000 |
| FVC%/D_LCO_% | 1.29(1.09-1.66) | 0.83(0.73-0.93) | .000 |
| LVEF (%) | 67.00 (62.00-71.00) | 67.00 (63.00-72.00) | .694 |
| LVFS (%) | 37.00 (34.00-41.00) | 37.00 (33.00-41.00) | .297 |
| pH | 7.43±0.03 | 7.42±0.03 | .079 |
| PaO_2_ (mmHg) | 71.10±14.41 | 72.12±15.36 | .178 |
| PaCO_2_ (mmHg) | 38.30 (35.10-42.25) | 39.00 (36.00-42.60) | .065 |
| PO_2_(A-a) (mmHg) | 27.30 (20.30-34.70) | 26.00 (19.45-33.35) | .179 |
| Leukocyte count (×10^9^/L) | 6.34 (5.12-8.03) | 6.64 (5.21-8.39) | .146 |
| Neutrophil count (×10^9^/L) | 4.18 (3.20-6.03) | 4.25 (3.12-5.90) | .809 |
| Platelet count (×10^9^/L) | 173.00 (135.00-209.00) | 188.00 (152.00-234.00) | .000 |
| Hemoglobin (g/L) | 134.66±18.12 | 137.30±15.36 | .034 |
| Albumin (g/L) | 39.20 (36.55-41.60) | 40.40 (37.55-43.00) | .000 |
| Globulin (g/L) | 24.73±4.60 | 24.96±4.13 | .480 |
| ALT (IU/L) | 16.00 (12.00-26.00) | 17.00 (12.00-25.00) | .223 |
| AST (IU/L) | 20.00 (16.00-25.00) | 18.82 (15.00-24.00) | .021 |
| DBIL (μmol/L) | 4.53 (3.40-6.03) | 4.40 (3.27-6.00) | .372 |
| IBIL (μmol/L) | 6.90 (4.90-9.56) | 6.85 (4.60-9.15) | .339 |
| Creatinine (μmol/L) | 70.70 (60.61-81.70) | 67.33 (56.58-78.00) | .002 |
| BUN (mmol/L) | 5.16 (4.21-6.21) | 4.96 (4.01-6.12) | .069 |
| Cystatin C (mg/L) | 1.00 (0.88-1.16) | 0.96 (0.84-1.10) | .004 |
| D-dimer (ng/mL) | 370.00 (200.00-645.00) | 340.00 (220.00-585.00) | .932 |
| Mortality (%) | 23.5 | 7.6 | .000 |

**Notes:** Data are expressed as means ± standard deviation or median (interquartile range) or percentage.

**Abbreviations:** FEV_1_, forced expiratory volume in 1 second; FVC, forced vital capacity; VA, alveolar ventilation; D_LCO_, diffusing lung capacity for carbon monoxide; LVEF, left ventricular ejection fraction; LVFS, left ventricular fractional shortening; PaO_2_, partial pressure of oxygen in arterial blood; PaCO_2_, partial pressure of carbon dioxide in arterial blood; PO2_(A-a)_, alveolar-arterial oxygen gradient; ALT, alanine aminotransferase; AST, aspartate aminotransferase; DBIL, direct bilirubin; IBIL, indirect bilirubin; BUN, blood urea nitrogen.
